# Precision Postoperative Radiotherapy in Sinonasal Carcinomas after Endonasal Endoscopic Surgery

**DOI:** 10.3390/cancers13194802

**Published:** 2021-09-25

**Authors:** Juliette Thariat, Florent Carsuzaa, Pierre Yves Marcy, Benjamin Verillaud, Ludovic de Gabory, Francois Regis Ferrand

**Affiliations:** 1 Department of Radiation Oncology, Centre Francois Baclesse, 14000 Caen, France; 2 ARCHADE Research Community, 14000 Caen, France; 3 Laboratoire de physique Corpusculaire IN2P3/ENSICAEN/CNRS UMR 6534—Normandie Université, 14000 Caen, France; 4 Department of Oto-Rhino-Laryngology—Head and Neck Surgery, Centre Hospitalier Régional Universitaire, 86194 Poitiers, France; florent.carsuzaa@gmail.com; 5 Department of Radiology, Clinique du Cap d’Or, 83500 La Seyne-sur-mer, France; pierreyves.marcy@idi83.fr; 6 Department of Oto-Rhino-Laryngology—Head and Neck Surgery, Hôpital Lariboisière, 75010 Paris, France; benjamin.verillaud@aphp.fr; 7 Department of Oto-Rhino-Laryngology—Head and Neck Surgery, Centre Hospitalier Universitaire, 33000 Bordeaux, France; ludovic.de-gabory@chu-bordeaux.fr; 8 French Armed Forces Biomedical Research Institute, 91220 Brétigny sur Orge, France; francoisregisferrand@gmail.com; 9 Department of Oto-Rhino-Laryngology—Oncology, Institut Gustave Roussy, 94805 Villejuif, France

**Keywords:** cancer, sinonasal/nasal cavity/paranasal/sinus, surgery, endoscopic, radiotherapy

## Abstract

**Simple Summary:**

Sinonasal cancers are rare and heterogeneous tumors, mainly carcinomas, with essentially local evolution and a severe vital and functional prognosis. These tumors are more and more being treated in first intent by a mini-morbid endoscopic approach rather than open surgery as the cornerstone of curative treatment. Adjuvant radiotherapy remains necessary owing to non-optimal local control. This article describes the requirements of radiotherapy to ensure adequate delays, the potential of postoperative radiotherapy to increase local and distant disease control and to decrease morbidity further after mini morbid surgery and dose painting techniques, and reviews the criteria that lead to the choice of one technique over another.

**Abstract:**

Radiotherapy plays an important role in the treatment of sinonasal cancer, mainly in the adjuvant setting after surgical resection. Many technological approaches have been described, including intensity-modulated radiotherapy, concomitant chemoradiotherapy, charged particle therapy or combined approaches. The choice is based on general criteria related to the oncological results and morbidity of each technique and their availability, as well as specific criteria related to the tumor (tumor extensions, pathology and quality of margins). The aims of this review are: (i) to provide an overview of the radiotherapy techniques available for the management of sinonasal malignant tumors and (ii) to describe the constraints and opportunities of radiotherapy owing to the recent developments of endonasal endoscopic surgery. The indication and morbidity of the different techniques will be discussed based on a critical literature review.

## 1. Introduction

Sinonasal neoplasms represent 3% of head and neck cancers and a yearly incidence of 1/100,000 inhabitants in France. The paranasal sinuses harbor a wide variety of histologically distinct neoplasms. There are complex anatomical structures that lie in close proximity to critical structures such as optic nerves, orbits and their content (eye-ball, oculomotor muscles and nerves), optic chiasm, pituitary gland, internal carotid artery, cranial nerves and brain. The paranasal sinuses are characterized by a complex shape, air-soft tissues/mucosae-bone interfaces and are variably filled in with secretions. These characteristics are of special relevance for surgery and postoperative radiotherapy.

The majority of sinonasal neoplasms are very aggressive with 5-year survival rates of 30–60% and local recurrence as the main cause of death, but their patterns of spread and response rates to chemo- and radiotherapy vary widely [1,2]. Sinonasal neoplasms as well as the treatment of these neoplasms can lead to altered olfaction, taste, respiration, speech, vision and cosmesis. The REFCOR (Reseau d’Expertise Francais des Cancers ORL Rares) and EURACAN (European reference network for rare adult solid cancers) networks have been dedicated to improving the diagnosis and therapeutic management of sinonasal neoplasms for more than 2 decades and since 2011 for the International Rare Cancer Initiative (IRCI).

In contrast to conventional sites (pharynx and oral cavity), histologic subtypes are more varied. Squamous cell carcinomas represent 50% of sinonasal sites, intestinal-type adenocarcinomas (ITAC) 13%, mucosal melanomas 7%, olfactive neuroblastomas 7%, adenoid cystic carcinomas 7%, undifferentiated carcinomas 3% and other histologies 13% [2,3]. Oncologic outcomes also vary according to the pathology: 5-year survival rates are 50% for squamous cell carcinomas, 60% for ITAC, 30% for melanomas, 70% for olfactive neuroblastomas, 70% for adenoid cystic carcinomas and neuroendocrine carcinomas and 35% for undifferentiated carcinomas. Local relapse is the dominant pattern of failure for all histologies except melanomas, which mostly fail distantly. For example, crude local relapse rates of sinonasal squamous cell carcinomas yield 40% (5-year local failure rate of 50%), of which less than 20% are efficiently salvaged [4], and ipsilateral level Ib-IIa and upper retropharyngeal nodes are common failure sites for T3–4 cases.

**Figure 1:**
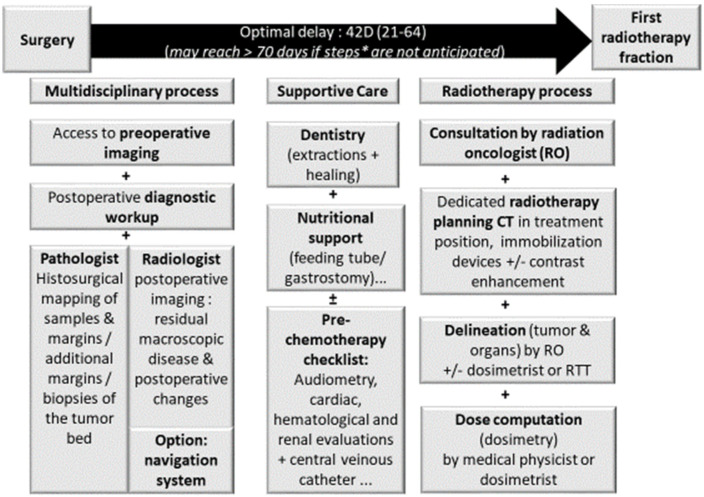
Prerequisite for adjuvant radiotherapy planning in sinonasal carcinomas. Legend: importance of time between surgery and radiotherapy on tumor control; RO: radiation oncologist.

Due to the non-specific symptoms, sinonasal tumors are often diagnosed late with locally advanced disease. While surgery is the mainstay of treatment in all histologies, achieving wide surgical resection with clear margins is a challenge. Resection is feasible in about 75% of sinonasal neoplasms [5,6] and only 53% of sinonasal squamous cell carcinomas [7,8]. Radiotherapy is thus usually indicated as a definitive exclusive treatment (generally with concomitant chemotherapy) when tumors are not resectable and as an adjuvant modality following surgery to optimize local control. Sinonasal tumors are indeed an example of multidisciplinarity and should be best performed at high volume centers [8,9] (Figure 1). The multidimensionality of the decision-making process is best demonstrated in borderline situations. Of those, orbital preservation best represents the compromise made to achieve acceptable oncological results while maximizing functional outcomes [10]. For example, induction chemotherapy and endoscopic-assisted surgery allowed orbital preservation in 76.6% of sinonasal squamous cell carcinomas; 96.0% of patients treated using an orbit-sparing approach maintained a functional eye after treatment [10].

We conducted a PubMed literature search using the terms «Radiotherapy», «Sinonasal OR sinus carcinoma», «adjuvant», «endoscopic surgery», «endonasal» and «chemotherapy», and selected the most meaningful articles based on our experience since all the reported articles come from small, retrospective and often monocentric series.

## 2. Toward Endoscopic Resection

Historical open surgery with transfacial and craniofacial resection was for a long time the only option for the surgical treatment of sinonasal tumors. These procedures have been associated with postoperative complication rates of 33–42% including 16.2% of neurologic complications and 3.5–4.5% mortality [11]. Surgery has evolved toward mini-morbid strategies [12,13], in particular, endoscopic approaches that have benefited from advances in instrumentation and optics since the 1990s. Endoscopic approaches were first described for ITAC, then for adenoid cystic carcinomas [14] and melanomas [15] and are now implemented regardless of histology. The advantage of endoscopic endonasal surgery is an optimal visualization of critical neurovascular structures and tumor attachment site allowing a more precise and targeted excision. Comparatively, endoscopic approaches have led to similar rates of positive margins of 21–24%, a major prognostic factor of survival (5y 25% with positive vs. 64% with negative margins, *p* < 0.0001) [16], to lower local failure rates (18% vs. 39%; *p* < 0.01) [17], even after adjustment on stage, and to lower complication rates (7% vs. 36%; OR = 3.5; *p* < 0.01). Endoscopic endonasal surgery also helps prevent complications and, in particular, cerebrospinal fluid leakage. When a dural resection is performed, a reconstruction with dural plasty is required. A reconstruction with a fascia lata, for example, can be used, covered by a vascularized flap, to facilitate healing. This vascularized flap can be a local flap (nasoseptal flap and turbinate flap) in the majority of cases, a regional flap (temporal fascia flap) less frequently or a free flap (forearm flap and anterolateral thigh flap) in cases of extended resections. Postoperative radiotherapy does not seem to be associated, on a small series, with failed CSF leak repair [18].

Finally, endoscopic endonasal surgery has consistently shown similar efficiency in tumor control and less morbidity, leading to reduced hospital stay compared with open surgery [16,19,20], although a surgical learning curve is observed [21,22]. Advances in in-room imaging using surgical navigation systems can help surgeons peroperatively [23] and might further improve the rate of safe margins, thus improving oncologic outcomes.

Endoscopic endonasal resection led to piecemeal resection of soft tissue fragments, or bone when needed, which may measure a few millimeters only. Each of these fragments is typically annotated using a standardized nomenclature and oriented by the surgeon [24]. Doing so, the pathologist can assess safe tissue fragments, tumor sub-volumes, quality of margins and additional margins.

Transition toward endoscopic approaches has had an impact on radiotherapy practice in ITAC [25]. The low morbidity of endoscopic approaches can be combined with modern irradiation modalities to promote safe, minimally invasive and maximally effective therapeutic options [26]. However, it is sometimes difficult to pinpoint the degree of extension in key anatomical structures due to sinonasal inflammation, tissue remodeling, angiogenesis and the specific behavior of the tumor. Preoperative imaging overestimates T stages in 76% of adenocarcinomas [27] while agreement is good between surgeon and pathologist. Moreover, the piecemeal resection can be misleading for the location of tumor extensions and margins, which determine the radiotherapy dose, and toxicities are cumulative between surgery and radiotherapy [28]. These findings point to the opportunity to improve patient care and avoid therapeutic escalation.

Based on differences in relapse patterns, postoperative radiotherapy target volumes may be adapted on histology in addition to stage to achieve reduction of radiation-induced morbidity by further exploiting the potential of endoscopic surgery.

## 3. Radiotherapy

### 3.1. Indication and Techniques

Patients with contraindications to surgery, either due to patient or tumor resectability, should receive concurrent chemoradiation. In the postoperative setting, all patients, except those with pT1 tumors involving the infrastructure only and with negative margins [29], benefit from adjuvant radiotherapy with differential magnitude of benefit across histologies [2]. Data are lacking after endoscopic surgery to modify expert recommendation based on mostly retrospective series [2,30]; furthermore, definition of margin is less clear with endoscopic procedures, and tumor dissemination may sometimes be an issue when en bloc resection cannot be achieved. Altogether, waiting for available new data reinforces the indication of adjuvant radiotherapy post endoscopic surgery with the same indication as that of a post open surgery.

Local control is dose-dependent (requiring 60–66 Gy) but technically challenging due to close proximity of critical organs and accompanying toxicity (which is also dose-dependent with dose > 45 Gy).

Technological advances have made minimally morbid radiotherapy possible. Integration of computer science in radiotherapy and advanced linear accelerator technologies in the 1990s have led to more conformal radiotherapy, i.e., intensity-modulated radiotherapy (IMRT). This has been achieved by means of computer- and multi-leaf collimator-guided photon fluence modulation to achieve steep dose gradients. The IMRT transition was primarily driven by the improvement in radiation dose distribution with IMRT compared to conventional two-dimensional (2D) and tridimensional conformal (3D) techniques [31]. As IMRT enables delivery of adequate doses to sinonasal tumors [32] while minimizing the dose to nearby critical organs [33], benefits in terms of local control and toxicities were expected, and this technique became the standard of care.

Charged particle therapy, which includes definitive/postoperative proton therapy (or definitive carbon ion therapy), is another recognized technique for the treatment of sinonasal neoplasms [34], but available machines remain rare.

### 3.2. Local Control with IMRT

With IMRT, 2- and 5-year local control rates are in the order of 80% and 50–70% for sinonasal tumors undergoing postoperative radiotherapy (+/−20% depending on histology) [35,36,37]. Most series are retrospective and consist of 50–200 patients. IMRT (including volumetric arc therapy) has consistently shown better 3-year locoregional recurrence-free survival rates by 20–30% compared with 3D conformal RT for carcinomas (89% vs. 60%; *p* = 0.035 [31], 85% vs. 65%, *p* = 0.02) [36,38]. For inoperable T4 paranasal sinus and skull-base tumors, the steep dose-gradient between tumor and normal tissue is even more advantageous, given the crucial need to maintain dose intensity to the tumor [39]. Some series also suggest improved survival with IMRT (60% vs. 72%; *p* = 0.02) while minimizing toxicities [40].

### 3.3. Toxicities Induced by IMRT

Similar or lower rate of acute toxicities not reaching significance have been observed with IMRT compared to 3D radiotherapy [38]. Fewer [6] (by about 20% compared with 3D radiotherapy) or less severe late toxicities have been reported [36,38]. Severe late radiation-induced toxicities have decreased in rate from about 50% of patients in the 1960s, 35–40% in the 1980s, and 15% in the 2000s and severe (grade 3+) to less debilitating (grade 2 or higher), yet bothersome symptoms. Historical series of conventional radiotherapy showed unilateral visual loss of up to 64% and bilateral blindness of about 2% [41]. IMRT significantly reduces the high doses to optic structures [42]. Subsequently, early IMRT studies showed major reduction of toxicities. While early IMRT series may have underestimated toxicity rates [43,44,45,46,47], other series report grade ≥ 2 late toxicities in the order of 28% [36]. Grade 2+ visual deterioration is reported in about 12–20% of patients [36,38,48,49,50]; 63% impaired olfactory function [50]; ear loss in 8–46% [5,51,52]); hormonal deficiencies in 22–62% including hypopituitarism in 24% [50,53]; cognitive dysfunction in up to 77% affecting short-term memory and speech; and fatigue in 18% [54,55]. According to Hamilton et al., sinonasal neoplasms are associated with significant symptoms during RT including pain, mucositis and dysgeusia [56].

Dose-response associations have been found between visual acuity impairment and the optic nerve and between fatigue and the temporal lobes after IMRT for sinonasal cancer but not for hypopituitarism or impaired olfactory function [50,57], possibly due to prescription dose to tumor nearby exceeding threshold dose for the given organ. Moreover, 11% of patients had structural abnormalities in irradiated areas of the brain, and impaired cognitive function was present in 63% of patients. Limited experience also suggests that quality of life (QoL) may return to pretherapeutic levels after 12 months using IMRT [36]. Impaired cognitive function as well as fatigue and insomnia were affected the most in QoL analyses [50]. Grade 3+ toxicity seems to be more likely in T4 tumors in relation to tumor stage and extensions, where compromise on tumor control may not be acceptable [36,50]. However, there seems to exist some latitude to apply stronger dose constraints to organs without jeopardizing tumor control [50].

Currently, the postoperative bed is usually irradiated at a similar high dose level without modulation on piecemeal analysis. IMRT may further allow selective dose escalation of RT to further improve outcomes. It is therefore advisable to better define, thanks to the clinico-radiological pair, the sinonasal subunits invaded by the tumor. In the meantime, some anatomical structures, for instance, involved in swallowing as late radiation-associated dysphagia, may often occur [30,58], which can be spared by dose reduction.

### 3.4. Toxicities Induced by Proton Therapy or Charged Particle Therapy

Charged particle therapy may better spare the organs at risk than IMRT [37,59,60,61,62] or may be used as an adjunct [52]. Despite increased sensitivity to anatomy, tumor and cavity filling change during radiotherapy of sinonasal neoplasms with protons versus photons, and organs at risk appear to receive smaller doses with proton therapy than IMRT [63] using adaptive replanning [64]. This is, however, dependent on the extent and bilaterality of tumors and may be best assessed by provisional dosimetry. Moreover, one risk of extreme conformality is geometric miss and unusual local relapse by unintentional sparing of tumor spread pathways (excess of leptomeningeal relapses have been observed after proton therapy due to better brain/meningeal sparing) [61,65].

Similar trends are observed with passive scattering compared multifield-optimized intensity-modulated proton therapy (IMPT) [37]. Limited experience with IMPT suggests that 24% experienced acute grade 3 toxicities, and 6% experienced late grade 3 toxicities (osteoradionecrosis, vision loss, soft-tissue necrosis and soft tissue fibrosis) [37]. Complication probability modelling (NTCP) can be used to inform patients of their likely outcomes.

## 4. Precision Radiotherapy

### 4.1. Definition of Irradiated Volumes and Histology-Specific Radiotherapy Tumor Volumes and Margins

Margins are added around the gross tumor (seen clinically before and during surgical resection and on imaging before resection) to account for microscopic tumor extensions along anatomic paths and across surrounding structures, so as to avoid a relapse. Tumor aggressiveness/invasiveness and patterns spread locally and into nodes depending on histology. Therefore, margins are tailored to anatomical barriers, nearby critical organs and histology. They are usually adapted to the risk of harboring microscopic tumor and to the probabilistic risk of tumor cell density, which is related to the risk of local relapse. Two dose levels may be defined centrifugally to account for a high risk or a moderate-to-low risk around the gross tumor. In the postoperative setting, the virtual gross tumor volume is first reconstructed to represent the postoperative tumor bed and delineated on the radiotherapy planning scanner (Figure 1). Then, 3–10 mm high-risk margins are added around the postoperative tumor bed and 5–10 mm moderate-to-low risk margins are added around the high-risk margins, typically for squamous cell carcinomas. These are adapted to the primary site of involvement and quality of margins. They are also adapted in case of perineural invasion (for example, for adenoid cystic carcinomas), lymphovascular emboli and other poor prognosis factors.

Similarly, the risk of nodal involvement varies with histology. Therefore, in case of N0 disease, nodal irradiation is also histology-specific [66] and tailored both on previous prophylactic neck dissection. Nodal involvement requires therapeutic consideration in squamous cell carcinomas, neuroblastomas and undifferentiated carcinomas. N0 presentations usually mandate prophylactic irradiation of levels Ib, IIa and upper retropharyngeal nodes (at the palate plane) unless prophylactic neck dissection has been performed. By contrast, nodal failure is rare in ITAC, adenoid cystic carcinomas and melanomas. Prophylactic nodal irradiation can therefore be omitted. In patients with metastatic nodes at diagnosis, irradiation of involved nodal areas should be performed as well as irradiation of close nodal areas uni- or bilaterally, depending on whether the tumor is strictly lateralized or not.

Beam arrangement has significant influence on plan quality in IMRT [67] and should be used to design plans that are robust to anatomy changes and changes in cavity filling. This is not necessarily through the use of non-coplanar IMRT [68] as tumor coverage and comparable organ sparing are achieved with coplanar beams, which, on the other hand, reduce peripheral doses and positioning uncertainties [68,69].

Homogeneous dose distribution has been considered as a goal of radiotherapy plans for years [70]. However, irradiating a whole tumor with high tumoricidal doses of photons may be associated with a risk of unacceptable severe toxicity. Tumor heterogeneity, as defined by accurate histosurgical mapping of piecemeal endoscopic sinonasal resection, may be exploited to escalate the dose in high-risk tumor sub-volumes and de-escalate the dose in the other lower-risk sub-volumes. This process illustrates the concept of dose painting. Dose requirements are usually defined by the level of risk of relapse, which itself is correlated with tumor cell density. The prerequisites for dose painting are that the relation between tumor control and dose, the dose prescription function, the dose-enhancement ratio and the areas of different risk are well known, and that this complex highly-modulated dose distribution is reproducible [71,72,73,74]. Clinical experience with IMRT suggests that the dose can be escalated by 10–15% technically, which is consistent with macroscopic residual tumor and positive or negative status margins as defined by histosurgical mapping after endoscopic sinonasal resection. Larger dose escalation with very steep dose gradients may be difficult to achieve.

The presence of sinonasal cavities and their air, bone and mucosa interfaces disrupt the homogenous dose distribution. Interfaces create areas of dose build-up and back-scatter and usually require adequate dose calculation algorithms accounting for secondary particles, such as the Monte Carlo method [75]. A decrease in dose before and within the air cavities and an increase in dose were observed, and the changes in dose distribution appeared dependent on the cavity size and depth [75].

Additional technical radiotherapy challenges include the need for adaptive radiotherapy to adapt dose distribution to the changing anatomy during radiotherapy. Anatomy, tumor and cavity filling changes typically occur during radiotherapy of sinonasal neoplasms. They may influence dose coverage of tumor sub-volumes or doses to organs to a larger extent with proton therapy compared with IMRT [63]. Robust beam angle selection or daily adaptive therapy may be needed to increase plan robustness to anatomical and positional uncertainties and to open the possibility of using improved and more conformal field arrangements [64].

Furthermore, as the human response to ionizing radiation (IR) varies among individuals [76], radiosensitizing tests are currently developed, in the SANTAL GORTEC 2016-02 trial (NCT02998385), in order to further adapt dosimetry on functional testing as a surrogate of individual radiosensitivity. Germline genetics are also being explored through genome-wide association study (GWAS) or more selective gene-defect approaches. Nevertheless, even with this promising future adaptation, late toxicities will remain meaningful issues, and follow-up programs are required [77].

### 4.2. Radiotherapy Planning after Endoscopic Endonasal Surgery

Postoperative radiotherapy should be planned so as to deliver irradiation between 21 days (for resolution of acute inflammatory processes and scarring) and 64 days following resection radiotherapy, at the latest [78]. Data in common squamous cell carcinomas of head and neck cancers even state that this delay should not exceed 42 days.

It should be anticipated that it takes 3–4 h to delineate all the structures of interest and that this must be carried out on a scanner machine that has been specifically-calibrated for radiotherapy. Additionally, this scanner is performed in treatment position using immobilization devices that guaranty accurate repositioning and comfort and have been commissioned for radiotherapy. It takes about 2–3 days (low range) to compute an IMRT dose distribution in agreement with tumor and organ at risk dose volume constraints defined after the delineation step by a dosimetrist or medical physicist. Supportive care and dentistry (+/−extractions and healing of about 2 weeks for simple maxillary extractions) may also be necessary. Considering that about 50 patients have their 10–15 min irradiation for about 30 daily Monday–Friday fractions on each radiotherapy machine, adding one patient to this busy workflow should be anticipated (Figure 1). Altogether, the full workflow emphasizes the fact that the patient should meet a radiation oncologist, at the latest, 3–4 weeks before starting irradiation and, therefore, at the latest, about 2 weeks after surgery. This may imply that pathology reports have not been finalized, or that a multidisciplinary meeting has not been held or that the surgeon has not been able to see the patient postoperatively. Ignoring such constraints and postponing radiotherapy for logistic reasons (some independent of the radiotherapy department organization) may translate in poorer tumor control.

Postoperative radiotherapy planning of sinonasal neoplasms, in its delineation step, requires virtual geolocation transfer of tumor from peroperative views to postoperative axial CT in daily radiotherapy practice. A second step is the determination of the dose to be delivered to parts of the operative bed. This dose depends on the risk of relapse, which itself depends on residual tumor cell density, and is assumed to correlate with the quality of margins. Residual macroscopic tumor will receive 70–74 Gy, microscopically positive margins 64–66 Gy, dubious or short margins 54–60 Gy and safe margins 45–54 Gy. These dose levels are thus highly dependent on the quality of pathologic analysis based on peroperative assessment, report and granularity of tissue resection and sampling by surgeons and pathologists.

Compared to current IMRT practice, personalized dose-painting IMRT based on histosurgical mapping from endoscopic surgery is technically demanding. With complex sinonasal anatomy managed as piecemeal resection and, subsequently, with a high number of anatomic structures analyzed and involved, including an analysis of margins and additional margins or biopsies of the operative bed, this virtual geolocation of endoscopic views onto postoperative CT slices is delicate. It requires excellent anatomic knowledge of the sinonasal cavities, 3D reconstruction of tissue fragments into voxels of a postoperative CT and technically complex modulation of irradiation on these voxels. To be transferred accurately, new radiotherapy sub-volume definitions require that piecemeal resection reports include position, size, orientation of each individually resected fragment and spacing between fragments to not result in a significant loss of information and uncertainties in radiotherapy delineation.

We showed a threefold reduction of volumes receiving high dose using dose-painting IMRT (125 mL vs. 41 mL), which resulted in larger volumes receiving low dose (133 mL vs. 189 mL). It is important to understand that de-escalation using dose-painting IMRT does not aim at omitting irradiation on the sinonasal cavities but rather to tailor the dose to the level of risk to the quality of margins assessed on each small endoscopically resected tissue fragment. However, due to the lack of specific sinonasal atlases identifying endoscopic resection fragments, the anatomic uncertainties and the level of confidence of using histosurgical mapping by radiation oncologists may limit the use of dose-painting IMRT in daily practice [79]. The multistep multidisciplinary approach requires training and should become more systematic [79]. Two trials are currently investigating de-escalation in high-dose volumes. The “SinocaRT” trial is a French phase II trial of dose-painting IMRT vs. standard IMRT. An American trial (NCT01586767) currently investigates whether proton therapy would result in equivalent or improved local control rate with similar or lower toxicity compared to IMRT, in the treatment of locally advanced sinonasal malignancy.

**Figure 2:**
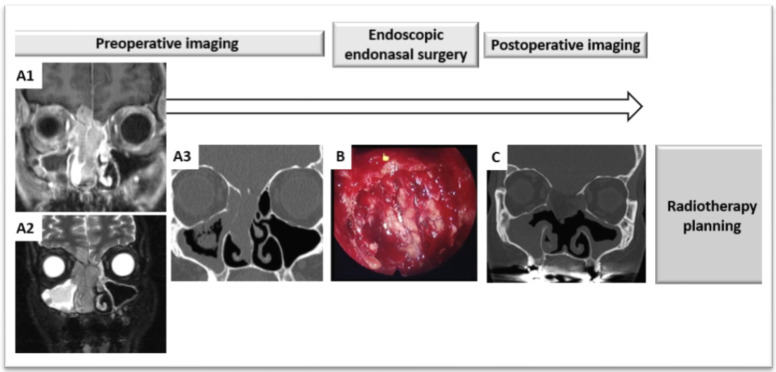
Perspectives of radiotherapy after endoscopic endonasal resection; a mini morbid multimodal strategy. Assessment of disease and therapeutic steps before radiotherapy planning in an olfactive neuroblastoma of the ethmoid. (**A1**): preoperative T1-gadolinium enhanced MR of the tumor, frontal view. (**A2**): preoperative T2 MR of the tumor, frontal view. (**A3**): preoperative CT of the tumor, frontal view. (**B**): endoscopic view of the tumor following resection. (**C**): postoperative CT at day 20 following tumor resection and base of skull reconstruction with abdominal fat flap, showing proper closure and no residual tumor.

**Figure 3:**
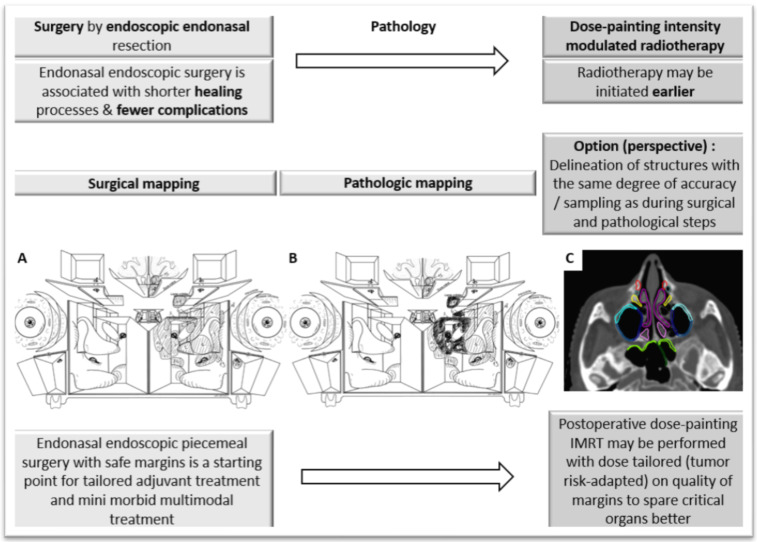
Perspectives of radiotherapy after endoscopic endonasal resection; a mini morbid multimodal strategy. Endonasal endoscopic resection of an adenocarcinoma of the ethmoid. (**A**,**B**): Pathologic mapping: of samples and margins using Bastier’s anatomical diagram for sinonasal malignant tumor resection/additional margins/biopsies of tumor bed on pathological report; grey-dashed grey areas representing safe margins, and black structures representing involved structures among resected structures [24]. (**C**): Accurate surgical sampling, peroperative sample annotation and localization of samples on operative report. Dashed areas representing resected structures.

**Figure 4:**
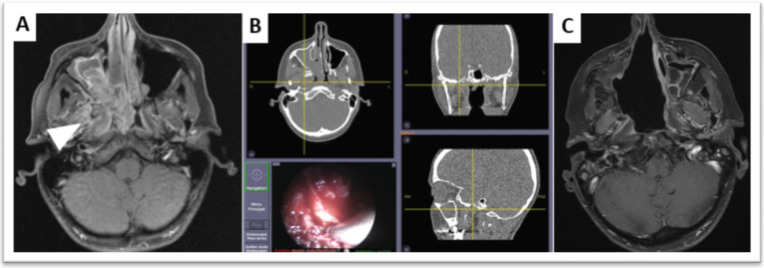
Surgical excision of an adenoid cystic carcinoma of the right nasal fossa using a surgical navigation system. (**A**): pre-operative contrast-enhanced T1-weighted MRI displays a right nasal fossa tumor invading the root of the pterygoids and the pterygoid muscles (arrowhead). (**B**): intraoperative computer-assisted surgical navigation system (DigiPointeur^®^, Collin, France) helps to correlate endoscopic and CT visualization of the tumor extension within the pterygoid muscles. (**C**): contrast-enhanced T1-weighted MRI confirms that the patient is free of local recurrence after 14 years of follow-up.

Improvements to facilitate dose-painting IMRT might come from optimized reporting during the endoscopic surgery procedure. A surgical navigation system using a digital pointer on 3D images may be exploited further by radiation oncologists when planning postoperative radiotherapy. If images and pointing information can be stored, these images may be co-registered and fused with postoperative radiotherapy planning CT [23]. 3D tumor reconstruction and printing might also be another option. It has been used for educational purposes with students or as some information aid for patients [80]. It could provide a postoperative basis to relocate positive margins on the tumor anatomy preoperatively. However, this may be time consuming for the surgeons and pathologists. Virtual transfer from a virtual 3D tumor to a postoperative scanner may not be more easily interpreted than with the standard report-based method or the pointed images from a navigation system used during endoscopic surgery. There are considerable knowledge gaps in these processes and their application to radiotherapy, and investigations are warranted (Figure 2, Figure 3 and Figure 4).

## 5. Combined Systemic Treatments

There are only grade C recommendations for use of chemotherapy or other systemic treatments in the setting of localized sinonasal neoplasm, with stronger evidence with concomitant use of chemotherapy with radiotherapy as adjuvant or exclusive treatment, while most teams advocate induction chemotherapy in case of unresectable or borderline resectable sinonasal neoplasms [81]. Anecdotally, preoperative radiotherapy, rather than postoperative radiotherapy, is consistently in time proposed by some teams with the aim to increase resectability in borderline resectable tumors (such as squamous cell carcinomas with orbital extension theoretically requiring exenteration), or to improve the quality of margins [82,83].

Options of systemic treatment in combination with radiotherapy are currently limited to cisplatin, which is used as a radiosensitizer (by creating DNA adducts in addition to DNA strand breaks from radiotherapy, thereby compromising repair) rather than a drug expected to have histology-specific response. This is the aim of the SANTAL GORTEC 2016-02 trial (NCT02998385), which investigates the role of concomitant cisplatin with IMRT (or proton therapy) in all the carcinoma subtypes with the exception of squamous cell carcinomas.

However, histology-specific systemic treatments may be developed based on experience in the metastatic or induction setting. Indeed, response to induction chemotherapy has been shown to be a surrogate marker for disease control by surgery and radiation and seems to predict radiotherapy response [81]. Concurrent chemoradiation by itself increases locoregional control in squamous cell cancer of the head and neck [84], which is particularly important in sinonasal localization, for which risk of local relapse is close to 30% [81]. Intestinal-type adenocarcinoma carrying a functional p53 protein may be treated with preoperative cisplatin, 5-fluorouracil and leucovorin [85]. Sinonasal undifferentiated carcinoma [86], neuroendocrine carcinoma [87] and high-grade olfactive neuroblastomas [88] are other types of sinonasal chemosensitive tumor, for which induction chemotherapy is often used to counterbalance the severe prognosis; however, owing to their scarcity, the added value of the platinum-based chemotherapy and the type of chemotherapy (i.e., with or without etoposide) have not been clearly defined yet. Therefore, multimodality trials are ongoing to test the activity of induction chemotherapy followed by locoregional curative treatment [81]. Whether surgery may be avoided after response to induction chemotherapy is debated; some teams advocate the demonstrated role of surgery in bad prognosis tumor in order not to omit it in the multimodal approach, while others highlight that definitive CRT after response to chemotherapy results in improved survival compared with those who undergo definitive surgery even followed by radiotherapy [86].

Chemotherapy might thus be maintained in the concomitant setting, provided that cumulative toxicity is acceptable. Genetic aberrations provide data that can help or fine-tune classification and provide molecular targets for treatment with specific inhibitors specific to sinonasal neoplasm histologies. More options for personalized treatment of sinonasal cancer patients are now opening up [89].

## 6. Conclusions

The poor prognosis of locally advanced sinonasal carcinoma has been largely improved since the development of transnasal endoscopic surgery and conformal radiation procedures, and further progress is still ongoing while multimodal approaches need the support of a strong cooperation capacity between physicians to fulfill quality of care.

Limitations of this review are essentially linked to heterogeneity and scarcity of data collected from small, often monocentric and retrospective series.

In this review, we outlined the great potential of neuronavigation techniques and post-operative imaging to achieve a better local control and decrease toxicity when combined with dose-painting IMRT.
